# Pharmacogenomic biomarkers of ACE inhibitor–induced cough in a multi-ethnic UAE cohort

**DOI:** 10.3389/fphar.2025.1655617

**Published:** 2025-09-25

**Authors:** Sahar M. Altoum, Zeina N. Al-Mahayri, Lubna Q. Khasawneh, Mais N. Alqasrawi, Lilas Dabaghie, Dana Hamza, Bassam R. Ali

**Affiliations:** ^1^ Department of Genetics and Genomics, College of Medicine and Health Sciences, United Arab Emirates University, Al-Ain, United Arab Emirates; ^2^ Department of Biomedical Sciences, College of Health Sciences, Abu Dhabi University, Al-Ain, United Arab Emirates

**Keywords:** hypertension, ACE inhibitors, cough, UAE, pharmacogenomics and personalized medicine, ACE I/D polymorphism, *BDKRB2*, *KCNIP4*

## Abstract

**Background and objectives:**

Angiotensin-converting enzyme inhibitors (ACEIs) are widely used to manage hypertension and cardiovascular diseases. However, dry cough is a common side effect, affecting 5%–35% of patients and often leading to discontinuation. This study aimed to investigate genetic variants involved in ACEI-induced cough and ACE plasma levels in UAE multi-ethnic hypertensive patients.

**Method:**

The study cohort was pragmatically selected from the larger EmHeart Study (n = 900), a UAE-based pharmacogenomic initiative. Patients prescribed ACEIs were screened for inclusion. This multi-center, retrospective exploratory study involved genotyping 107 patients treated with ACEIs, including n = 35 in the cough group and n = 72 in the non-cough group. Variants of *ACE*; rs1799752 I/D, *BDKRB2*; rs1799722 (C>T), and *KCNIP4*; rs7675300 (C>A), rs1495509 (T>C), rs7661530 (T>C), and rs16870989 (T>A) were genotyped using standard technologies. A sandwich ELISA was done to investigate the ACE plasma levels in our cohort.

**Results:**

We found that the *ACE* rs1799752 I/D genotype in the over-dominant model, was statistically significantly associated with ACEI-induced cough (p = 0.046) after adjusting for gender. Similarly, the T/T genotype of the *KCNIP4* rs7661530 (T>C) variant was associated with significantly higher risk of cough compared to the combined C/C and T/C genotypes (p = 0.035). In contrast, the variants *BDKRB2* rs1799722 (C>T), *KCNIP4* rs7675300 (C>A), rs1495509 (T>C), and rs16870989 (T>A) were not significantly associated with ACEI-induced cough in our study. Moreover, ACE plasma levels were significantly lower in the cough group compared to the non-cough group (p = 0.0014). Stratified analysis by rs1799752 I/D genotypes revealed a significant difference within the I/D genotype (p = 0.0061), with higher levels in the non-cough group. No significant differences were found for the D/D or I/I genotypes.

**Conclusion and limitations:**

Our data showed a significant association between ACEI-induced cough and the ACE rs1799752 I/D genotype, as well as lower ACE plasma levels in the cough group. This is the first study in the UAE and Middle East to report such findings and include all these variants in a single analysis. Although the sample size is small, our results contribute cumulative evidence on the genetic predisposition to ACEI-induced cough among hypertensive patients.

## Introduction

Angiotensin-converting enzyme inhibitors (ACEIs) are considered the standard treatment for hypertension and have been widely used for many years. They exert their effects by inhibiting the angiotensin-converting enzyme (ACE), which prevents the conversion of angiotensin I to the vasoconstrictor angiotensin II. Moreover, ACEIs inhibit the release of the aldosterone hormone, leading to increased sodium and water excretion. These actions lead to a reduction in blood pressure (Altoum et al., 2023; Herman et al., 2025).

Despite their effectiveness, ACEIs are linked to side effects that may limit their use (Manoharan, 2023). The most frequently observed side effect is a dry cough, affecting between 5% and 35% of treated patients (Hallberg et al., 2017). The likelihood of experiencing an ACEI-induced cough is higher among females, individuals with respiratory conditions such as asthma, and the elderly (Ding et al., 2020; Hallberg et al., 2017). This cough is characterized by dryness and a tickling or scratching sensation in the throat. It may occur as early as after the first dose or gradually develop over weeks to months. Although the cough usually presents with mild to moderate severity, there are instances when it becomes severe enough to necessitate the discontinuation of the medication. While the cough may linger for up to 3 months after ceasing ACEIs, it typically resolves within one to 4 weeks (Dicpinigaitis, 2006).

The exact mechanism behind the cough induced by ACEIs remains elusive. However, it is suspected that several mediators, including the vasodilator bradykinin (BK), play a role in triggering this type of cough. BK is a vasodilator that reduces blood pressure by binding to the Bradykinin Receptor B2 (BDKRB2). Moreover, BK can cause bronchoconstriction by prompting the production of other mediators, such as prostaglandins, histamine, and leukotrienes. Subsequently, BK is rapidly degraded into its inactive metabolites, primarily by ACE. Accordingly, when ACEIs are administered, they inhibit BK’s metabolism by blocking the ACE’s action. This inhibition leads to an accumulation of BK in respiratory tracts and binds to the BDKRB2 resulting in bronchospasm and cough (Altoum et al., 2023; Pinto et al., 2020).

Given that the occurrence of cough due to ACEIs is unpredictable, it is reasonable to suggest that ACEI-induced cough is influenced by one or more genetic factors (Borghi et al., 2023). The *ACE;*rs1799752 (Insertion/Deletion), *BDKRB2*;rs1799722 (C>T), and variants located on Potassium Voltage-Gated Channel Interacting Protein 4 (*KCNIP4*); rs145489027 (G>A), rs7675300 (C>A), rs1495509 (T>C), rs7661530 (T>C), rs16870989 (T>A), and rs6838116 (A>C) variants have been identified as key genetic contributors involved in the intricate genetic basis of ACEI-induced cough in some populations (Mosley et al., 2016; Mu et al., 2019; Mukae et al., 2000). The *ACE;* rs1799752 variant located within intron 16 of the *ACE* is characterized by the Insertion (I) and/or Deletion (D) of 287 base pairs “Alu repeat” and is known to affect the ACE serum levels. The (D/D) carriers have higher ACE serum levels in contrast to those with heterozygous (I/D) and homozygous insertion (I/I) carriers, with intermediate and low ACE serum levels, respectively. The latter two groups are known to accumulate BK, which ACE normally degrades. Consequently, patients in these groups are at a higher risk of experiencing cough from ACEIs compared to those with the (D/D) genotype (Abbas et al., 2012; Mu et al., 2019).

Moreover, a study has shown a significant correlation between the rs1799722 (C>T) variant located in the *BDKRB2* promoter region and ACEI-induced cough. *BDKRB2*: rs1799722 T/T genotype carriers were found to be more prone to developing cough than C/C genotype (Mukae et al., 2000). Finally, a genome-wide association study (GWAS) identified *KCNIP4* single nucleotide polymorphisms (SNPs) within intron 4 to be associated with ACEI-induced cough (Mosley et al., 2016). Consequently, studies have documented the correlation between these genetic variants and ACEI-induced cough in various populations.

Despite the fact that ACEI-induced cough is not considered a severe side effect, in some cases, it results in the discontinuation of ACEIs (Borghi et al., 2023). It is well-known that the leading risk factor for heart failure, coronary heart disease, stroke, and end-stage renal disease is hypertension. Consequently, discontinuing hypertension treatment might seriously affect the patients (Rohman et al., 2018). Accordingly, we sought to investigate the genetic factors that might predict the occurrence of ACEI-induced cough. Although pharmacogenomics in cardiovascular disease research has been conducted in the United Arab Emirates (UAE) (Al-Mahayri et al., 2022; Alqasrawi et al., 2024; 2025; Khasawneh et al., 2024), studies on the pharmacogenomics of ACEI-induced cough have never been reported in the UAE or any other Middle Eastern populations.

Our current study aimed to assess the association between various genetic variants suggested by previous research in other populations with ACEI-induced cough among adult hypertensive patients in the UAE. The selected variants included *ACE*; rs1799752 I/D, *BDKRB2*; rs1799722 (C>T), and *KCNIP4*; rs7675300 (C>A), rs1495509 (T>C), rs7661530 (T>C), and rs16870989 (T>A). Additionally, we explored the relationship between ACE plasma levels and cough induction, correlating these findings with the genotypes present in the *ACE* I/D variant.

In addition, we aimed to examine the distribution of these variants across different ethnic groups, assessed linkage disequilibrium (LD) patterns, constructed haplotypes to explore combined genetic effects, and performed variant interaction analysis to evaluate potential gene-gene interactions. To our knowledge, this is the first study in the UAE and the Middle East to report such findings and the first to include all these variants in a single analysis.

## Methods

### Study design and patients

This is a retrospective multicenter study conducted at four recruitment sites, Tawam Hospital, The Heart Medical Center, and Mediclinic Al-Ain Hospital in Al-Ain, UAE, and Burjeel Day Surgery Center in Abu Dhabi, UAE, from October 2022 to October 2023. The study was conducted in accordance with the Declaration of Helsinki and approved by the Department of Health-Abu Dhabi for the Data from the UAEU (DOH/CVDC/2020/1187), (DOH/CVDC/2021/1519), (DOH/CVDC/2022/1458), (DOH/CVDC/2023/1952) (MCME.CR.213.MAIN.2021), and (SNA/FA/2020-14). Our sample was drawn from the EmHeart Study, the first pharmacogenomic implementation initiative conducted in the UAE, which focuses on integrating pharmacogenomic data into cardiovascular drug prescribing to improve safety and efficacy. The original cohort included 900 patients. We screened the entire cohort to identify those who had been prescribed ACEIs. The inclusion criteria included adults aged 18 years and above with hypertension who are currently taking ACEIs for at least 1 year or had used ACEIs before and discontinued due to ACEI-induced cough. The Exclusion criteria included (1) Those who didn’t receive ACEIs, (2) Those who had discontinued ACEI therapy due to side effects unrelated to cough, such as headache, dizziness, or angioedema, (3) Patients who were currently using ACEIs but for less than 1-year, (4) Patients with diseases associated with chronic cough including severe asthma, COPD, and GERD, (5) patients with any secondary cause of hypertension, and (6) patients who disagree to give blood for the study. A group of 107 hypertensive adult patients signed an informed consent form to participate in this study. Two distinct groups of patients were recruited: a non-cough group comprised of 72 patients who received any type of ACEIs for at least 1 year without experiencing ACEI-induced cough. The second group, the cough group, comprised 35 patients who had used ACEIs and developed ACEI-induced cough.

### Data collection

**FIGURE 1:**
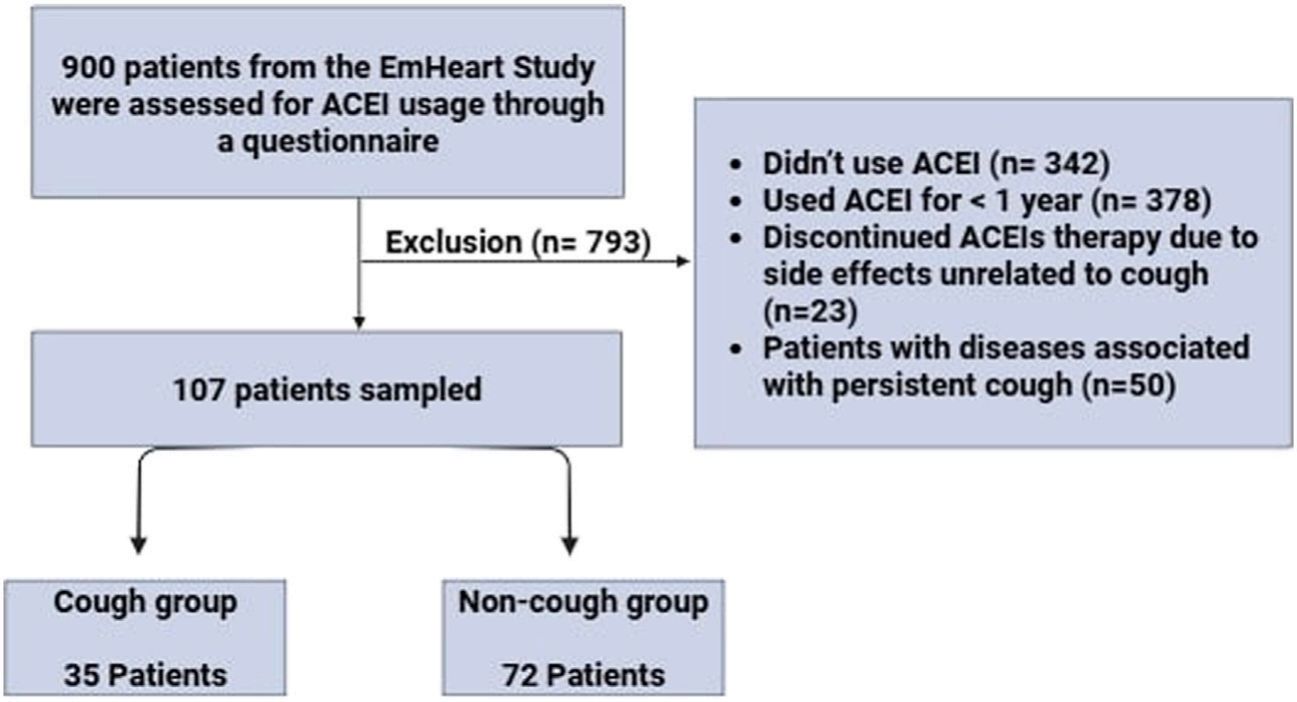
The flow of patient recruitment and the selection process within the study.

Data were collected from the patient’s medical records, including demographic (age, gender, ethnicity), health behaviours (smoking status), and concomitant medications. To gather detailed information on ACEI usage and associated cough, we first conducted a thorough review of each patient’s electronic medical records (EMR) for physician documented evidence of ACEI-induced cough, including its onset timing and severity. Following this, we directly contacted patients to confirm ACEI use and further clarify the occurrence and characteristics of the cough. A structured questionnaire was developed to support this process. It initially confirmed whether the patient had received any ACEIs and then assessed the presence of ACEI-induced cough. The questionnaire included targeted questions on the reason for ACEI discontinuation, time of cough onset relative to ACEI initiation, frequency and severity of coughing episodes, including daytime vs nighttime patterns, as well as the specific ACEI type used. Figure 1 represents the flow of participant recruitment and the selection process within the study.

### Blood sampling and DNA extraction

Blood samples were obtained from the peripheral vein and were collected in 3 mL vacuum tubes containing EDTA (BD Inc.). Genomic DNA was extracted from whole blood using QIAamp^®^ DNA kit (Qiagen, Germany) according to the manufacturer’s protocol. The quality and quantity of DNA were determined using a Nanodrop One Spectrophotometer (Thermo Fisher Scientific, United States). Plasma samples were prepared by centrifuging 500 mL of whole blood at 2,500 rpm for 10 min at room temperature, and the resultant plasma was then stored at −80 °C for future use.

### Amplification of the *ACE*, *BDKRB2,* and *KCNIP4* variants

Primers for amplifying specific gene regions were designed using the Primer3 website (https://primer3.ut.ee/), and the primer sequences are provided in Table 1. The PCR reaction was carried out in a total volume of 25 μL, consisting of combining 13.5 µL of DNAse free water, 1x of 10x PCR Buffer, 1x of 5x Q-Solution, 200 µM of dNTPs, 1.5 units of Taq DNA Polymerase, 100–200 pmol of the forward primer, 100–200 pmol of the reverse primer and 296–350 ng of DNA. Amplification was performed using a SimpliAmp thermal cycler (ThermoFisher Scientific, Waltham, Massachusetts, U.S.A.) over 32 cycles, each cycle included pre-denaturation at 95 °C for 5 minutes, denaturation at 95 °C for 45 s, annealing at either 57 °C or 60 °C for 45 s, extension at 72 °C for 45 s, and a final extension at 72 °C for 7 min. PCR products were separated using electrophoresis on a 1.5% agarose gel stained with ethidium bromide and visualized under a UV illuminator.

**TABLE 1:** List of variants and their PCR oligonucleotide primers.

Gene and Target	Forward primer	Reverse primer	Ann. Temp (°C)	Product size (bp)
*ACE* rs1799752 (g.63488543_63488544ins-11TGAGACGGAGTCTCGCTCTGTCGCCCATACAGTCACTTTT)	5′-CTG​GAG​ACC​ACT​CCC​ATC​CTT​TCT-3′	5′-GAT​GTG​GCC​ATC​ACA​TTC​GTC​AGA​T-3′	60	190 (D), 490 (I)
*BDKRB2* rs1799722 (g.96204802C>T)	5′- GGG​CTA​CGC​AAA​CAT​GGA​AA-3′	5′- AGT​TTG​TCC​TCC​CAG​CAG​AG-3′	57	399
*KCNIP4* rs7675300 (g.21383914C>A)	5′- TTT​GCA​TGG​AGG​GGA​TCA​CT-3′	5′- GGC​TGT​GAG​GTA​GGA​CTG​AG-3′	57	456
*KCNIP4* rs1495509 (g.21391993T>C)	5′-TCC​CCT​GCA​ATC​ACA​TTC​CT-3′	5′- TGC​CAG​AGT​TCC​CTT​CCA​TT-3′	60	371
*KCNIP4* rs7661530 (g.21349637T>C)	5′-ATG​TGT​CAT​TCA​GCA​GCG​TC-3′	5′- GAG​AAT​GCT​AGC​CCT​TGT​GC-3′	60	380
*KCNIP4* rs16870989 (g.21385141T>A)	5′-GGG​CGC​TTT​GTC​TCT​TTT​CT-3′	5′- TTT​ATC​CCA​CCT​CCT​GCT​GG-3′	60	375

Ann. Temp: Annealing Temperature, bp: base pairs, ACE: angiotensin converting enzyme, BDKRB2: Bradykinin Receptor B2, KCNIP4: Potassium Voltage-Gated Channel Interacting Protein 4, D: deletion, I: insertion.

### Identification of genetic variants

**FIGURE 2:**
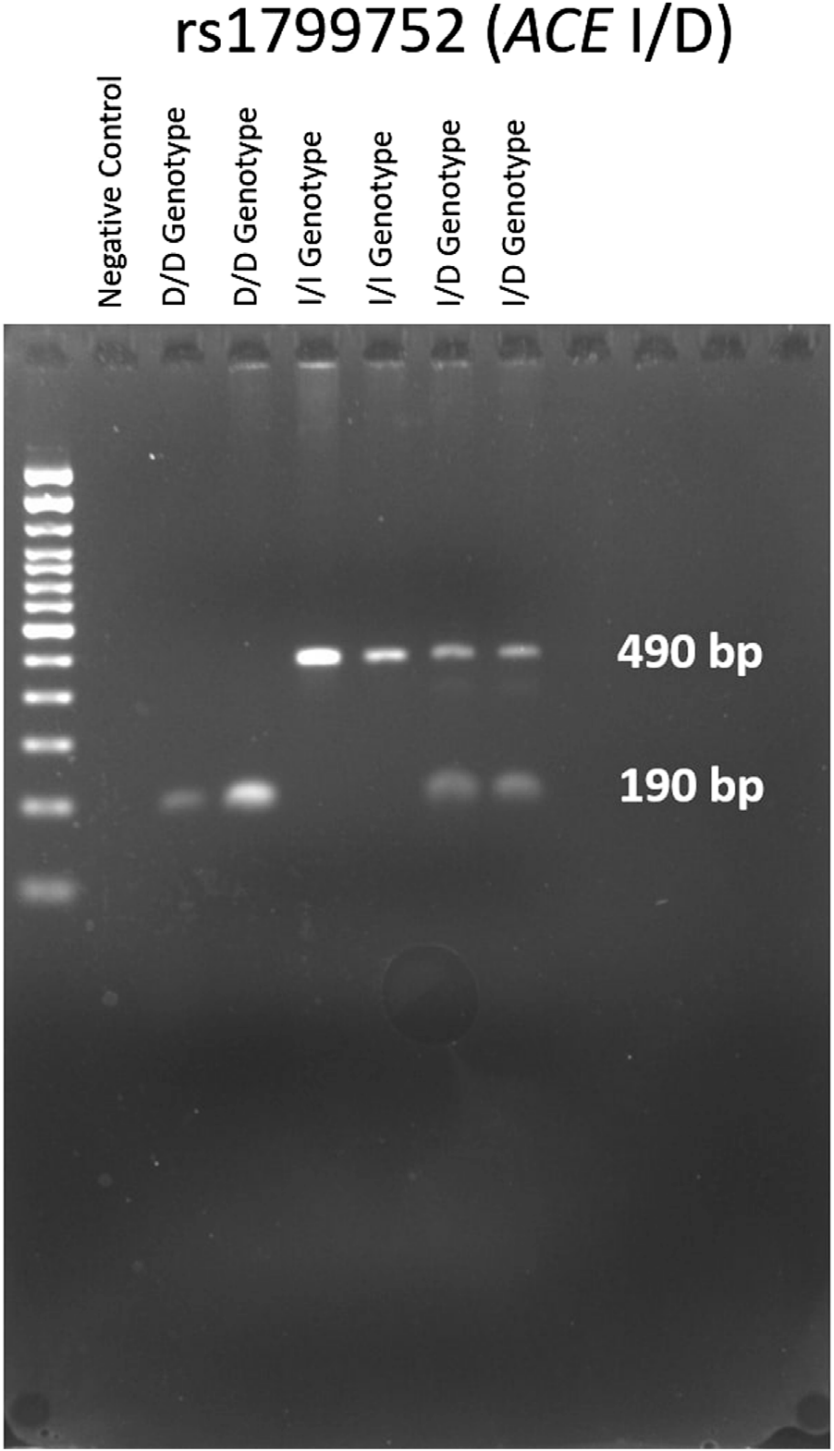
Representative agarose gel results for *ACE*; rs1799752 (I/D) genotypes (D/D, I/I and I/D).

The genotypes of the *ACE*; rs1799752 I/D variant were determined through gel electrophoresis of the PCR products, as illustrated in Figure 2. The electrophoresis resulted in a 190 bp product corresponding to the D allele and a 490 bp product corresponding to the I allele. In heterozygote samples, both 490 and 190 bp bands were observed. Genotypes for *BDKRB2*; rs1799722 (C>T), and *KCNIP4*; rs7675300 (C>A), rs1495509 (T>C), rs7661530 (T>C), and rs16870989 (T>A) were identified by sanger sequencing using the BigDye Terminator Cycle Sequencing Kit (Applied Biosystems, Waltham, Massachusetts, United States). Sequencing analysis was conducted with the 3130xl Genetic Analyzer (Applied Biosystems, Waltham, Massachusetts, United States). The results were processed using Chromas software (Technelysium, Australia).

### Measurement of ACE plasma levels

Sandwich Enzyme-Linked Immunosorbent Assay (ELISA) was employed to measure ACE protein levels in plasma samples from various patients using the Human ACE ELISA Kit (ab263889) (Abcam, Cambridge, United Kingdom) strictly following the manufacturer’s provided instructions. To ensure the accuracy of the ELISA results and minimize potential bias, the assay was performed on plasma samples collected exclusively from patients who were not currently taking ACEIs or had not taken the medication within 5 days prior to blood sample collection. This precaution was taken to avoid any possible interference from the medication on the assay outcomes. Additionally, to enhance the reliability and precision of the results, each plasma sample was tested in quadruplicate, with four separate wells assigned to each sample.

### Variants distribution within ethnicities

To explore potential ethnicity-specific patterns, we performed a stratified analysis of genotype frequencies across the main ethnic groups represented in our cohort. Ethnicity information was initially collected from medical records and then confirmed directly with patients to ensure accuracy.

### Linkage disequilibrium (LD)

To investigate the non-random association between alleles at different loci, we conducted LD analysis among the *KCNIP4* rs7675300 (C>A), rs1495509 (T>C), rs7661530 (T>C), and rs16870989 (T>A) variants.

### Haplotype analysis

Haplotype analysis was performed to investigate the combined effect of the *KCNIP4* variants (rs7675300, rs1495509, rs7661530, and rs16870989) on ACEI-induced cough.

### Variant interaction analysis

To evaluate potential synergistic effects among genetic variants we conducted variant interaction analysis involving *ACE* rs1799752 (I/D), *BDKRB2* rs1799722 (C>T), and *KCNIP4* rs7675300 (C>A).

### Statistical analysis

Statistical analyses were conducted using SNPStats and SPSS version 29.0.2.0 (SPSS Inc., US) (Solé et al., 2006). ELISA results were processed with GraphPad Prism version 10.2.2 (GraphPad Software, Boston, Massachusetts, United States). Categorical variables and Hardy-Weinberg equilibrium (HWE) were analyzed using the Chi-square (χ2) test. Numerical data were presented as means with standard deviations. Odds ratios with 95% confidence intervals [OR; 95% CI] were computed to determine associations, considering p < 0.05 statistically significant. For ELISA results, the Mann-Whitney test was used to compare total ACE plasma levels between the two study groups and within the study groups for each *ACE*-rs1799752 I/D genotype, and the Kruskal–Wallis test was applied to analyze differences in ACE plasma levels across the three *ACE* I/D genotypes, with p < 0.05 considered statistically significant.

Chi-square tests were performed to assess the significance of variants distribution across different ethnic groups, with p < 0.05 considered statistically significant. Further, adjusted residuals greater than +2 or less than −2 was used to identify specific genotype-ethnicity associations. LD among *KCNIP4* variants was measured by calculating D′ values using the SHEsisPlus platform (Shen et al., 2016). Haplotype construction was carried out using the SNPStats software. Logistic regression analysis was performed to calculate the ORs and 95% CIs for each haplotype compared to the reference. Finally, variant interaction analysis was conducted to examine the collective impact of all variants on the risk of ACEI-induced cough.

## Results

### Patient’s baseline demographics and clinical characteristics

107 adult hypertensive patients were identified from the EmHeart study as ACEIs users and recruited for this study. The incidence of cough in our study was 32.7% (35 patients). The baseline characteristics and collected data, including demographics, comorbidities, and ACEIs type, are presented in Table 2. 73.8% of the participants were males, with no statistically significant difference in gender distribution between the cough and no-cough groups (P = 0.18). The average age across all patients was 53.79 (±11.13) years. Moreover, the participants represented a diverse ethnic background categorized into five distinct groups (Arabs, East Asians, Indians, Africans, and others). Coronary heart disease affected 48.6% of patients equally in both groups. Diabetes mellitus was slightly more common in the cough group (54.3%) than in the non-cough group (50%). Chronic kidney disease was the least prevalent, occurring in 5.6% of patients, with similar distribution in both groups.

**TABLE 2:** Patient baseline characteristics of ACEI-induced cough and non-cough groups.

Baseline characteristics	Total patients (n = 107)	Cough (n = 35)	Non-cough (n = 72)	P value
Age (years)				
	53.79 ± 11.13	53.37 ± 12.34	54 ± 10.57	0.78
Gender				
Male	79 (73.8%)	23 (65.7%)	56 (77.8%)	0.18
Female	28 (26.2%)	12 (34.3%)	16 (22.2%)	
Smoking				
	37 (34.6%)	11 (31.4%)	26 (36.1%)	0.633
Blood Pressure Reading				
Systolic pressure (mmHg)	129.98 ± 16.13	127.37 ± 16.82	131.25 ± 15.74	0.24
Diastolic pressure (mmHg)	78.51 ± 12	75.41 ± 12.3	79.97 ± 11.74	0.069
Ethnicity				
Arab	53 (49.5%)	17 (48.6%)	36 (50%)	0.89
East Asian	13 (12.1%)	3 (8.5%)	10 (13.9%)	0.53
Indians	38 (35.5%)	15 (42.9%)	23 (31.9%)	0.26
African	2 (1.87)	0	2 (2.8%)	1
Others	1 (0.93%)	0	1 (1.4%)	1
Comorbidities				
Hypertension	107 (100%)	35 (100%)	72 (100%)	NA
Coronary heart disease	52 (48.6%)	17 (48.6%)	35 (48.6%)	0.99
Chronic kidney disease	6 (5.6%)	2 (5.7%)	4 (5.6%)	1
Diabetes Mellitus	55 (51.4%)	19 (54.3%)	36 (50%)	0.67
ACEIs type				
Perindopril	52 (48.5%)	17 (48.6%)	35 (48.6%)	0.99
Lisinopril	46 (43%)	17 (48.6%)	29 (40.3%)	0.25
Ramipril	7 (6.5%)	0	7 (9.7%)	0.09
Enalapril	1 (1%)	1 (2.8%)	0	0.32
Captopril	1 (1%)	0	1 (1.4%)	1

^a^ p < 0.05 was considered significant. ACEIs: Angiotensin Converting Enzyme Inhibitors.

Regarding ACEIs usage among patients, perindopril was the most commonly prescribed ACEIs to 48.5% of the total population, with almost identical usage in both the cough (48.6%) and non-cough groups (48.6%). Lisinopril was the second most frequently used ACEIs, accounting for 43% of total usage, with a slightly higher proportion in the cough group (48.6%) than the non-cough group (40.3%). Ramipril was used by 6.5% of patients, all of whom were in the non-cough group. The least commonly prescribed ACEIs were Enalapril and Captopril, each used by only 1% of the participants, with Enalapril used exclusively in the cough group and Captopril in the non-cough group. Overall, there was no statistically significant difference in the distribution of ACEIs type among the two groups.

For patients in the cough group, information regarding the duration of ACEIs usage, onset of cough, and drug discontinuation is provided in Table 3. The duration of ACEIs usage varied, with the most common being 2–6 months and 1–7 weeks, each by 25.7% and 22.8% of the patients, respectively. A smaller percentage used ACEIs for longer durations. The onset of cough after starting ACEIs varied, with the highest percentages reported within the first week, 28.5%, and from 1–7 weeks to 2–6 months, each 25.7%. The cough occurred mostly at night in 60% of the patients. A total of 94.3% discontinued ACEIs due to the tolerable nature of the cough, and in all cases (100%), the cough resolved within less than 1 week after discontinuation.

**TABLE 3:** Cough group ACEIs usage information.

Characteristics	Cough (n = 35)
Duration of ACEIs use	
1–7 weeks	8 (22.8%)
2–6 months	9 (25.7%)
7–11 months	7 (20%)
1–5 years	7 (20%)
>5 years	4 (11.4%)
Time from start of ACEIs to occurrence of cough	
<1 week	10 (28.5%)
1–7 weeks	9 (25.7%)
2–6 months	9 (25.7%)
7–11 months	4 (11.4%)
1–5 years	3 (8.5%)
Time of cough	
All the day	14 (40%)
Night	21 (60%)
ACEIs discontinuation	
Discontinued	33 (94.3%)
Continued	2 (5.7%)
Drug discontinuation decision (n = 33)	
Patient’s self-own	5 (15.1%)
Under Physicians supervision	28 (84.8%)
Cough disappearance after ACEIs discontinuation (n = 33)	
<1 week	33 (100%)

ACEIs: Angiotensin Converting Enzyme Inhibitors.

### Association of *ACE*, *BDKRB2*, and *KCNIP4* alleles and genotypes with ACEI-induced cough

The genotype frequencies of *ACE*-rs1799752 I/D, *BDKRB2*-rs1799722 (C>T), and *KCNIP4*- rs7675300 (C>A), rs1495509 (T>C), rs7661530(T>C), and rs16870989 (C>A) variants were consistent with HWE in both cough and non-cough groups (p > 0.05). Detailed results are presented in Supplementary Table S1. Tables 4–9 present the crude analysis, as well as the analysis after adjusting for the confounding factor (gender) for the variants. The analysis was conducted using various genetic models, including codominant, dominant, recessive, and over-dominant models.

**TABLE 4:** Association between *ACE* rs1799752 I/D variant and ACEI-induced cough.

Model	Genotype	Total (n = 107)	Cough (n = 35)	Non-cough (n = 72)	OR (95% CI)^a^	P-value^a^	OR (95% CI)^b^	P-value^b^
Codominant	D/D	44 (41%)	11 (31.4%)	33 (45.8%)	1.00	0.15	1.00	0.13
	I/D	41 (38%)	18 (51.4%)	23 (31.9%)	2.35 (0.94–5.89)		2.52 (0.99–6.45)	
	I/I	22 (21%)	6 (17.1%)	16 (22.2%)	1.12 (0.35–3.59)		1.25 (0.38–4.06)	
Dominant	D/D	44 (41%)	11 (31.4%)	33 (45.8%)	1.00	0.15	1.00	0.11
	I/D + I/I	63 (59%)	24 (68.6%)	39 (54.2%)	1.85 (0.79–4.32)		2.01 (0.84–4.81)	
Recessive	D/D + I/D	85 (79%)	29 (82.9%)	56 (77.8%)	1.00	0.54	1.00	0.62
	I/I	22 (21%)	6 (17.1%)	16 (22.2%)	0.72 (0.26–2.05)		0.77 (0.27–2.19)	
Over-dominant	D/D + I/I	66 (62%)	17 (48.6%)	49 (68.1%)	1.00	0.053	1.00	**0.046**
	I/D	41 (38%)	18 (51.4%)	23 (31.9%)	2.26 (0.99–5.16)		**2.34 (1.01–5.41)**	
Allele frequency	D	129 (0.6)	40 (0.57)	89 (0.62)	1.21 (0.68–2.17)	0.61	---	---
	I*	85 (0.4)	30 (0.43)	55 (0.38)				

OR: odds ratio; CI: confidence interval; *: Risk allele.

^a^ crude analysis; ^b^Adjusted for gender analysis. p < 0.05 was considered significant.

Significant values are indicated by Bold font.

**TABLE 5:** Association between *BDKRB2* rs1799752 (C>T) variant and ACEI-induced cough.

Model	Genotype	Total (n = 107)	Cough (n = 35)	Non-cough (n = 72)	OR (95% CI)^a^	P-value^a^	OR (95% CI)^b^	P-value^b^
Codominant	C/C	37 (35%)	9 (25.7%)	28 (38.9%)	1.00	0.29	1.00	0.37
	C/T	53 (49%)	21 (60%)	32 (44.4%)	2.04 (0.80–5.18)		1.92 (0.75–4.91)	
	T/T	17 (16%)	5 (14.3%)	12 (16.7%)	1.30 (0.36–4.69)		1.27 (0.35–4.61)	
Dominant	C/C	37 (35%)	9 (25.7%)	28 (38.9%)	1.00	0.17	1.00	0.22
	C/T + T/T	70 (65%)	26 (74.3%)	44 (61.1%)	1.84 (0.75–4.49)		1.74 (0.70–4.29)	
Recessive	C/C + C/T	90 (84%)	30 (85.7%)	60 (83.3%)	1.00	0.75	1.00	0.77
	T/T	17 (16%)	5 (14.3%)	12 (16.7%)	0.83 (0.27–2.58)		0.85 (0.27–2.65)	
Over-dominant	C/C + T/T	54 (51%)	14 (40%)	40 (55.6%)	1.00	0.13	1.00	0.17
	C/T	53 (49%)	21 (60%)	32 (44.4%)	1.87 (0.83–4.26)		1.77 (0.77–4.06)	
Allele frequency	C	127 (0.59)	39 (0.56)	88 (0.61)	1.25 (0.7–2.23)	0.54	---	---
	T*	87 (0.41)	31 (0.44)	56 (0.39)				

OR: odds ratio; CI: confidence interval; *: Risk allele.

^a^ crude analysis; ^b^Adjusted for gender analysis. p < 0.05 was considered significant.

**TABLE 6:** Association between *KCNIP4* rs7675300 (C>A) variant and ACEI-induced cough.

Model	Genotype	Total (n = 107)	Cough (n = 35)	Non-cough (n = 72)	OR (95% CI)^a^	P-value^a^	OR (95% CI)^b^	P-value^b^
Codominant	C/C	50 (47%)	13 (37.1%)	37 (51.4%)	1.00	0.23	1.00	0.16
	C/A	43 (40%)	15 (42.9%)	28 (38.9%)	1.52 (0.63–3.71)		1.75 (0.69–4.39)	
	A/A	14 (13%)	7 (20%)	7 (9.7%)	2.85 (0.84–9.67)		3.23 (0.92–11.34)	
Dominant	C/C	50 (47%)	13 (37.1%)	37 (51.4%)	1.00	0.16	1.00	0.097
	C/A + A/A	57 (53%)	22 (62.9%)	35 (48.6%)	1.79 (0.78–4.09)		2.05 (0.87–4.84)	
Recessive	C/C + C/A	93 (87%)	28 (80%)	65 (90.3%)	1.00	0.15	1.00	0.13
	A/A	14 (13%)	7 (20%)	7 (9.7%)	2.32 (0.74–7.24)		2.45 (0.77–7.74)	
Over-dominant	C/C + A/A	64 (60%)	20 (57.1%)	44 (61.1%)	1.00	0.7	1.00	0.55
	C/A	43 (40%)	15 (42.9%)	28 (38.9%)	1.18 (0.52–2.68)		1.29 (0.56–2.98)	
Allele frequency	C	143 (0.67)	41 (0.59)	102 (0.71)	1.72 (0.95–3.12)	0.1	---	---
	A*	71 (0.33)	29 (0.41)	42 (0.29)				

OR: odds ratio; CI: confidence interval; *: Risk allele.

^a^ crude analysis; ^b^Adjusted for gender analysis. p < 0.05 was considered significant.

**TABLE 7:** Association between *KCNIP4* rs1495509 (T>C) variant and ACEI-induced cough.

Model	Genotype	Total (n = 107)	Cough (n = 35)	Non-cough (n = 72)	OR (95% CI)^a^	P-value^a^	OR (95% CI)^b^	P-value^b^
Codominant	T/T	52 (49%)	15 (42.9%)	37 (51.4%)	1.00	0.5	1.00	0.38
	T/C	42 (39%)	14 (40%)	28 (38.9%)	1.23 (0.51–2.97)		1.35 (0.55–3.31)	
	C/C	13 (12%)	6 (17.1%)	7 (9.7%)	2.11 (0.61–7.34)		2.44 (0.68–8.74)	
Dominant	T/T	52 (49%)	15 (42.9%)	37 (51.4%)	1.00	0.41	1.00	0.3
	T/C + C/C	55 (51%)	20 (57.1%)	35 (48.6%)	1.41 (0.62–3.18)		1.56 (0.67–3.59)	
Recessive	T/T + T/C	94 (88%)	29 (82.9%)	65 (90.3%)	1.00	0.28	1.00	0.22
	C/C	13 (12%)	6 (17.1%)	7 (9.7%)	1.92 (0.59–6.22)		2.12 (0.64–6.99)	
Over-dominant	T/T + C/C	65 (61%)	21 (60%)	44 (61.1%)	1.00	0.91	1.00	0.82
	T/C	42 (39%)	14 (40%)	28 (38.9%)	1.05 (0.46–2.39)		1.10 (0.48–2.55)	
Allele frequency	T	146 (0.68)	44 (0.63)	102 (0.71)	1.44 (0.78–2.62)	0.3	---	---
	C*	68 (0.32)	26 (0.37)	42 (0.29)				

OR: odds ratio; CI: confidence interval; *: Risk allele.

^a^ crude analysis; ^b^Adjusted for gender analysis. p < 0.05 was considered significant.

**TABLE 8:** Association between *KCNIP4* rs7661530 (T>C) variant and ACEI-induced cough.

Model	Genotype	Total (n = 107)	Cough (n = 35)	Non-cough (n = 72)	OR (95% CI)^a^	P-value^a^	OR (95% CI)^b^	P-value^b^
Codominant	C/C	56 (52%)	16 (45.7%)	40 (55.6%)	1.00	0.11	1.00	0.082
	T/C	35 (33%)	10 (28.6%)	25 (34.7%)	1.00 (0.39–2.55)		1.02 (0.40–2.63)	
	T/T	16 (15%)	9 (25.7%)	7 (9.7%)	**3.21 (1.02–10.10)**		**3.55 (1.10–11.40)**	
Dominant	C/C	56 (52%)	16 (45.7%)	40 (55.6%)	1.00	0.34	1.00	0.3
	T/C + T/T	51 (48%)	19 (54.3%)	32 (44.4%)	1.48 (0.66–3.34)		1.55 (0.68–3.52)	
Recessive	C/C + T/C	91 (85%)	26 (74.3%)	65 (90.3%)	1.00	**0.035**	1.00	**0.025**
	T/T	16 (15%)	9 (25.7%)	7 (9.7%)	**3.21 (1.08–9.54)**		**3.52 (1.16–10.65)**	
Over-dominant	C/C + T/T	72 (67%)	25 (71.4%)	47 (65.3%)	1.00	0.52	1.00	0.52
	T/C	35 (33%)	10 (28.6%)	25 (34.7%)	0.75 (0.31–1.81)		0.75 (0.31–1.82)	
Allele frequency	T*	67 (0.31)	28 (0.40)	39 (0.23)	1.79 (0.98–3.28)	0.07	---	---
	C	147 (0.69)	42 (0.60)	105 (0.73)				

OR: odds ratio; CI: confidence interval; *: Risk allele.

^a^ crude analysis; ^b^Adjusted for gender analysis. p < 0.05 was considered significant.

Significant values are indicated by Bold font.

**TABLE 9:** Association between *KCNIP4* rs16870989 (T>A) variant and ACEI-induced cough.

Model	Genotype	Total (n = 107)	Cough (n = 35)	Non-cough (n = 72)	OR (95% CI)^a^	P-value^a^	OR (95% CI)^b^	P-value^b^
Codominant	T/T	50 (47%)	13 (37.1%)	37 (51.4%)	1.00	0.31	1.00	0.2
	T/A	44 (41%)	16 (45.7%)	28 (38.9%)	1.63 (0.67–3.93)		1.84 (0.74–4.59)	
	A/A	13 (12%)	6 (17.1%)	7 (9.7%)	2.44 (0.69–8.60)		2.91 (0.79–10.66)	
Dominant	T/T	50 (47%)	13 (37.1%)	37 (51.4%)	1.00	0.16	1.00	0.097
	T/A + A/A	57 (53%)	22 (62.9%)	35 (48.6%)	1.79 (0.78–4.09)		2.05 (0.87–4.84)	
Recessive	T/T + T/A	94 (88%)	29 (82.9%)	65 (90.3%)	1.00	0.28	1.00	0.22
	A/A	13 (12%)	6 (17.1%)	7 (9.7%)	1.92 (0.59–6.22)		2.12 (0.64–6.99)	
Over-dominant	T/T + A/A	63 (59%)	19 (54.3%)	44 (61.1%)	1.00	0.5	1.00	0.41
	T/A	44 (41%)	16 (45.7%)	28 (38.9%)	1.32 (0.58–2.99)		1.42 (0.62–3.27)	
Allele frequency	T	144 (0.67)	42 (0.60)	102 (0.71)	1.62 (0.89–2.94)	0.15	---	---
	A*	70 (0.33)	28 (0.40)	42 (0.29)				

OR: odds ratio; CI: confidence interval; *: Risk allele.

^a^ crude analysis; ^b^Adjusted for gender analysis. p < 0.05 was considered significant.

### ACE rs1799752 (I / D) variant

The genotypic and allelic frequencies of the *ACE* I/D variant are presented in Table 4. Among this variant, 31.4% were D/D, 51.4% were I/D, and 17.2% were I/I in the cough group. Among the non-cough, 46% were D/D, 32% were I/D, and 22% were I/I. The frequency of the I allele was 0.43 in the cough group and 0.38 in the non-cough group; however, there was no statistically significant association between the I/I genotype (p = 0.15) or the I allele (p = 0.61) and ACEI-induced cough. Nevertheless, after adjusting for gender, the over-dominant model showed the most statistically significant association with ACEI-induced cough, where the heterozygous I/D genotype is linked to a higher risk of ACEI-induced cough compared to the combined D/D and I/I genotypes, with an OR of 2.34 and p = 0.046.

### *BDKRB2* rs1799752 (C>T) variant

The genotypic and allelic frequencies of the *BDKRB2* rs1799752 (C>T) are presented in Table 5. Among this variant, 26% were C/C, 60% were C/T, and 14% were T/T in the cough group. Among the non-coughers, 39% were C/C, 44% were C/T, and 17% were T/T. The frequency of the T allele was 0.44 in the cough group and 0.39 in the non-cough group; however, there was no statistically significant association between the T allele (p = 0.54) or T/T genotype and ACEI-induced cough either in the crude analysis (p = 0.29) or after adjusting for gender (p = 0.37).

### *KCNIP4* rs7675300 (C>A) variant

The genotypic and allelic frequencies of the *KCNIP4* rs7675300 (C>A) are presented in Table 6. Among this variant, 37% were C/C, 43% were C/A, and 20% were A/A in the cough group. Among the non-coughers, 51% were C/C, 39% were C/A, and 10% were A/A. The frequency of the A allele was 0.41 in the cough group and 0.29 in the non-cough group; however, there was no statistically significant association between the A allele (p = 0.1) or A/A genotype and ACEI-induced cough either in the crude analysis (p = 0.23) or after adjusting for gender (p = 0.16).

### *KCNIP4* rs1495509 (T>C) variant

The genotypic and allelic frequencies of the *KCNIP4* rs1495509 (T>C) are presented in Table 7. Among this variant, 43% were T/T, 40% were T/C, and 17% were C/C in the cough group. Among the non-cough, 51% were T/T, 39% were T/C, and 10% were C/C. The frequency of the C allele was 0.37 in the cough group and 0.29 in the non-cough group; however, there was no statistically significant association between the C allele (p = 0.3) or C/C genotype and ACEI-induced cough either in the crude analysis (p = 0.5) or after adjusting for gender (p = 0.38).

### *KCNIP4* rs7661530 (T>C) variant

The genotypic and allelic frequencies of the *KCNIP4* rs7661530 (T>C) are presented in Table 8. Among this variant, 26% were T/T, 29% were T/C, and 45% were C/C in the cough group. Among the non-cough, 10% were T/T, 35% were T/C, and 55% were C/C. The frequency of the T allele was 0.40 in the cough group and 0.23 in the non-cough group. Although a higher frequency of the T allele was observed in the cough group compared to the non-cough group, indicating an increased risk of ACEI-induced cough for the T/T genotype (OR = 3.21), this result did not reach statistical significance in the codominant model (p = 0.11). However, the recessive model shows a statistically significant association between the T/T genotype and ACEI-induced cough in both the crude and adjusted analyses. In the crude analysis, patients with the T/T genotype have a significantly increased risk of ACEI-induced cough compared to those with the combined C/C and T/C genotypes, with an OR of 3.21 and a p = 0.035. Similarly, in the adjusted-to-gender analysis, the T/T genotype is associated with a significantly increased risk of cough, with an OR of 3.52 and a p = 0.025.

### *KCNIP4* rs16870989 (T>A) variant

The genotypic and allelic frequencies of the *KCNIP4* rs1495509 (T>C) are presented in Table 9. Among this variant, 37% were T/T, 46% were T/A and 17% were A/A in the cough group. Among the non-cough, 51% were T/T, 39% were T/A and 10% were A/A. The frequency of the A allele was 0.40 in the cough group and 0.29 in the non-cough group; however, there was no statistically significant association between the A allele (p = 0.15) or the A/A genotype and ACEI-induced cough either in the crude analysis (p = 0.31) or after adjusting for gender (p = 0.20).

### ACE plasma levels

**FIGURE 3:**
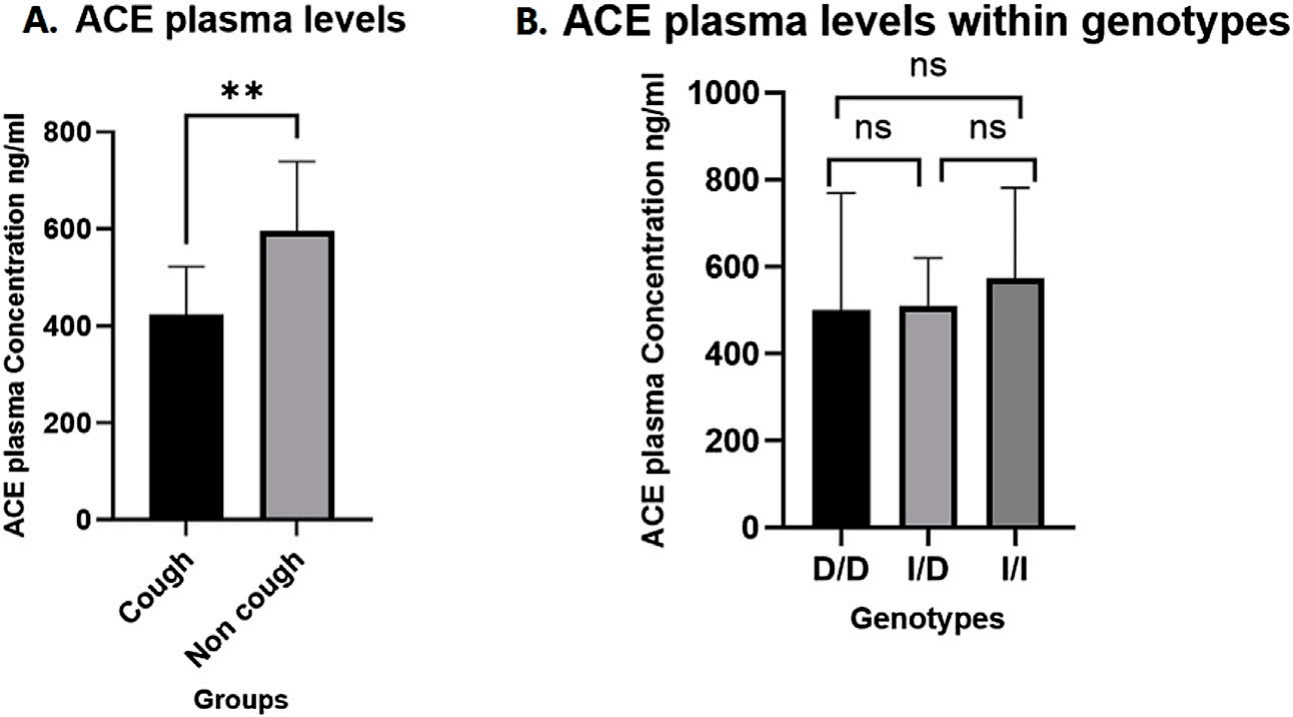
**(A)** Comparison of ACE plasma levels Between cough and non-cough groups. **(B)** Comparison ACE plasma levels across *ACE* I/D genotypes (D/D, I/D, and I/I genotypes). ** Indicates statistically significant difference (p < 0.05), ns: not significant.

**FIGURE 4:**
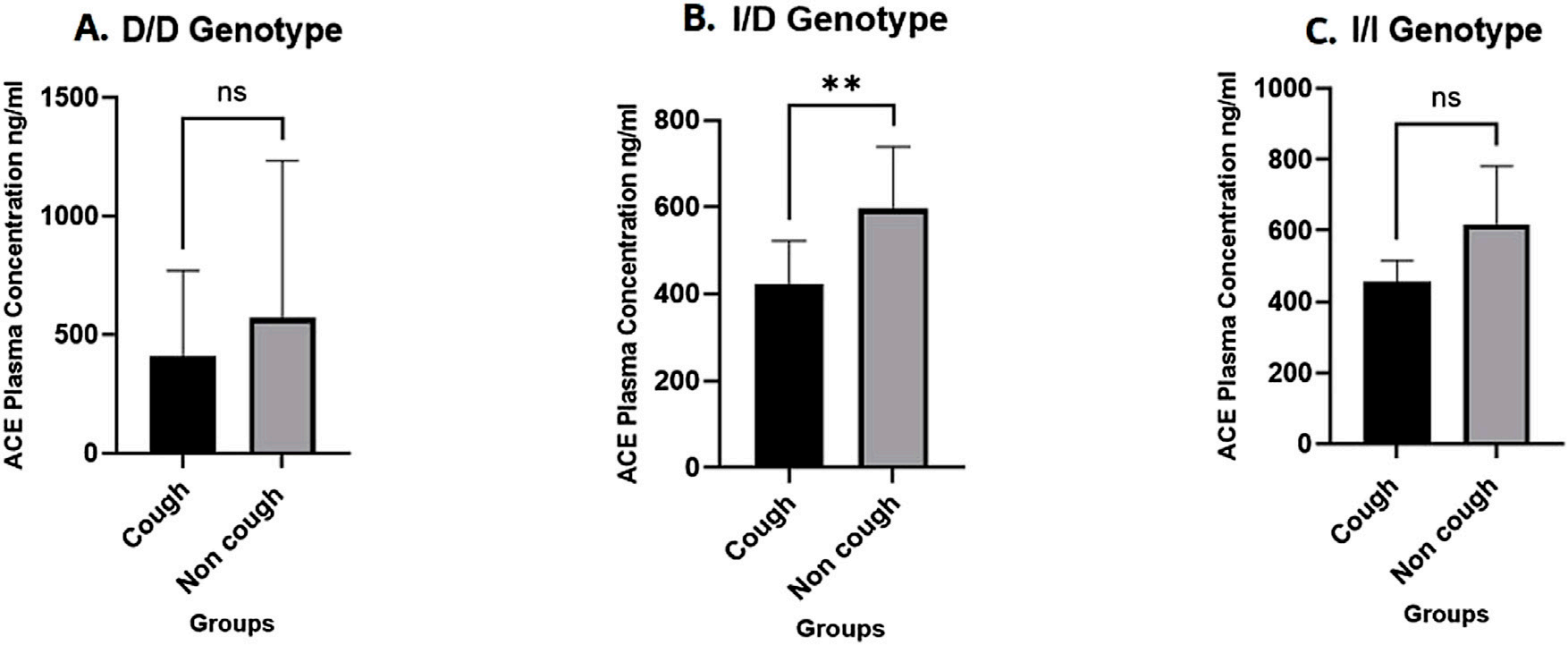
Comparison of ACE plasma levels by *ACE* I/D genotypes between the Cough and Non-cough Groups. **(A)** Comparison of ACE plasma levels within D/D genotype. **(B)** Comparison of ACE plasma levels within I/D genotype. **(C)** Comparison of ACE plasma levels within I/I genotype. ** Indicates statistically significant difference (p < 0.05), ns: not significant.

A total of 28 plasma samples were analysed using an ELISA kit, with 14 samples from each group (cough and non-cough), focusing on the distribution across the three rs1799752 *ACE* I/D genotypes. The median ACE plasma levels were significantly lower in the cough group compared to the non-cough group (423 ng/mL and 595.8 ng/mL, respectively; p = 0.0014, Figure 3A), suggesting a potential association between decreased ACE levels and the occurrence of ACEI-induced cough in the cough group. Furthermore, when stratified by the rs1799752 I/D genotypes, the median plasma levels for the D/D, I/D, and I/I genotypes were 499.9 ng/mL, 510.4 ng/mL, and 574.8 ng/mL, respectively. While there was a trend of higher levels in individuals with the I/I genotype compared to the D/D and I/D genotypes, the difference between the three genotypes was not statistically significant (p = 0.43) as shown in Figure 3B. Finally, comparing the plasma levels for each rs1799752 I/D genotype between the cough and non-cough groups, a statistically significant difference was found in the I/D genotype (p = 0.0061) where the cough group exhibited lower plasma levels of 437.8 ng/mL compared to the non-cough group 593.6 ng/ml as shown in Figure 4B. In contrast, no significant differences were observed for the D/D and I/I genotypes. For the D/D genotype in Figure 4A, the plasma levels were 408.1 ng/mL in the cough group and 569.8 ng/mL in the non-cough group, with p = 0.54. Similarly, for the I/I genotype in Figure 4C, the plasma levels were 455.4 ng/mL in the cough group and 616.8 ng/mL in the non-cough group, with a p = 0.19.

### Variants distribution within ethnicities

A total of 107 patients were included in the analysis representing diverse ethnic backgrounds. The majority were Arab (n = 53), followed by Indian (n = 38), East Asian (n = 13), African (n = 2), and other ethnicities (n = 1). The stratified analysis of genotype distributions across these groups was conducted, and the detailed results are presented in Supplementary Tables S2–S7.

For the *ACE* rs1799752 (I/D) variant (Supplementary Table S2), a p < 0.01 indicated a statistically significant difference in genotype distribution among ethnic groups, with the Arab group showing a notably higher-than-expected frequency of the D/D genotype (adjusted residual = + 4.4) while the Indian group showed a significantly lower-than-expected frequency of the same genotype (adjusted residual = - 4.4). In contrast, for the *BDKRB2* rs1799722 (C>T) variant (Supplementary Table S3), the p = 0.15 indicated no statistically significant difference in distribution across ethnicities.

For the *KCNIP4* rs7675300 (C>A) variant (Supplementary Table S4), a p = 0.04 indicated a statistically significant difference in genotype distribution across ethnic groups. The Arab group showed a higher-than-expected frequency of the C/C genotype (adjusted residual = + 3.2), while the Indian group showed a lower-than-expected frequency of the same genotype (adjusted residual = - 2.7). In contrast, the East Asian group had a higher-than-expected frequency of the A/A genotype (adjusted residual = + 3). Similarly, for *KCNIP4* rs1495509 (T>C) variant (Supplementary Table S5), a p = 0.04 indicated a statistically significant difference in genotype distribution across ethnic groups. The Arab group had a higher-than-expected frequency of the T/T genotype (adjusted residual = + 2.8), while the Indian group showed a higher-than-expected frequency of the T/C genotype (adjusted residual = + 2.5). For *KCNIP4* rs7661530 (T>C) variant (Supplementary Table S6), the p = 0.23 indicated no statistically significant difference in genotype distribution across ethnicities. Finally, for the *KCNIP4* rs16870989 (T>A) variant (Supplementary Table S7), a p = 0.04 indicated a statistically significant difference, with the Arab group showing a higher-than-expected frequency of the T/T genotype (adjusted residual = + 3.2), and the Indian group displaying a higher-than-expected frequency of the T/A genotype (adjusted residual = + 2.2).

### Linkage disequilibrium analysis

**FIGURE 5:**
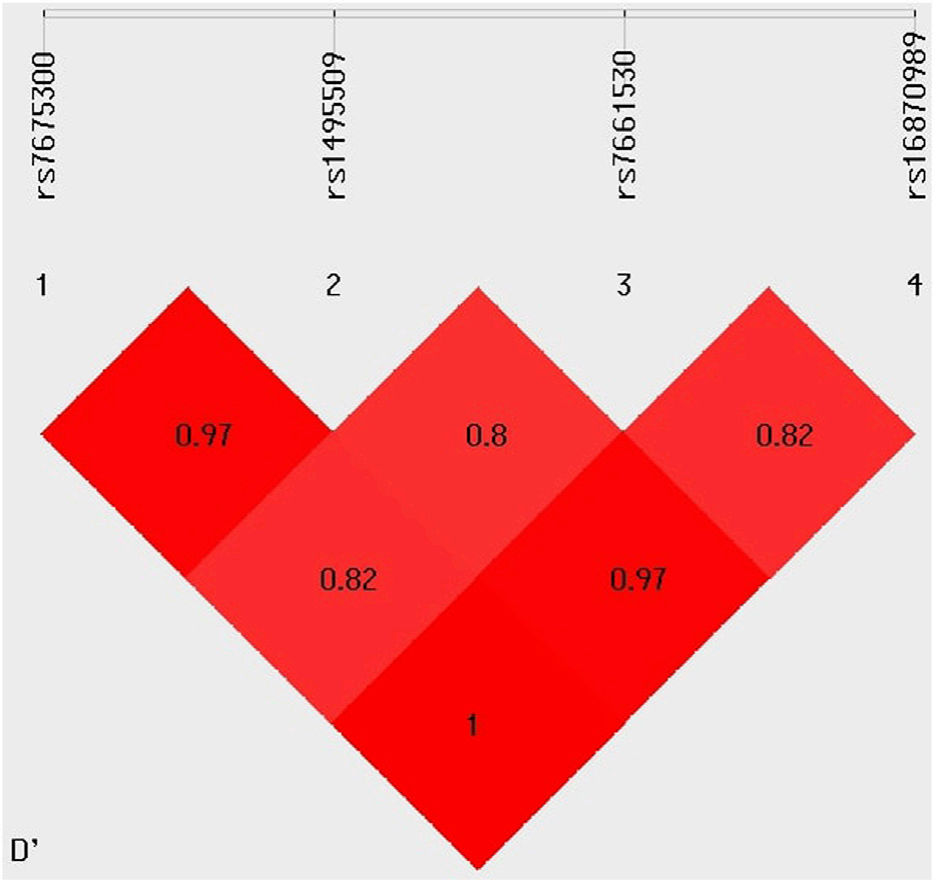
Linkage disequilibrium analysis of *KCNIP4* variants.

The LD analysis of four *KCNIP4* variants (rs7675300, rs1495509, rs7661530, and rs16870989) revealed strong non-random associations between them. The D′ values indicated a high degree of co-inheritance, with rs7675300 and rs16870989 showing complete LD (D' = 1), and rs7675300 and rs1495509 demonstrating very strong LD (D' = 0.97). Similar strong linkage was observed between rs1495509 and rs16870989, suggesting that these variants are frequently inherited together within the study population. These findings are presented in Figure 5.

### Haplotype association with ACEI-induced cough

Haplotypes were constructed based on the LD analysis. Haplotype 1 (CTCT), with a frequency of 0.6241, was the most prevalent in the study population and served as the reference. Although some haplotypes exhibited high ORs indicative of potential increased risk, none reached statistical significance. Additionally, the global haplotype association test yielded a p = 0.38, indicating no significant overall association between the haplotypes and ACEI-induced cough. The detailed results of this analysis are presented in Table 10.

**TABLE 10:** KCNIP4 Haplotype frequencies and their association with ACEI-induced cough.

Haplotype	rs7675300 (C>A)	rs1495509 (T>C)	rs7661530 (T>C)	rs16870989 (T>A)	Frequency	OR (95% CI)	P-value
1	C	T	C	T	0.6241	1.00	---
2	A	C	T	A	0.2698	1.58 (0.86–2.91)	0.14
3	A	C	C	A	0.0432	1.52 (0.33–6.94)	0.59
4	C	T	T	T	0.0394	2.61 (0.60–11.43)	0.21
5	A	T	C	A	0.0102	3.55 (0.22–58.09)	0.38
Rare	*	*	*	*	0.0133	5.12 (0.40–66.05)	0.21
Global haplotype association p-value: 0.38							

OR: odds ratio; CI: confidence interval.

### Variant interaction analysis

Due to high LD among the four *KCNIP4* variants, rs7675300 (C>A) was selected as a representative. The results, summarized in Table 11, show that the interaction between rs1799752 (I/D) and rs1799722 (C>T) had a p = 0.870, the interaction between rs1799752 (I/D) and rs7675300 (C>A) had a p = 0.841, and the three-way interaction among rs1799752 (I/D), rs1799722 (C>T), and rs7675300 (C>A) had a p = 0.965. These findings indicate no statistically significant synergistic effect of these variants on the risk of ACEI-induced cough.

**TABLE 11:** Variant interaction analysis between *ACE*, *BDKRB2*, and *KCNIP4* variants.

Variants interaction	B	p-value	OR (95% CI)
rs1799752 and rs1799722	−0.157	0.870	0.855 (0.131–5.580)
rs1799752 and rs7675300	−0.178	0.841	0.837 (0.147–4.755)
rs1799752, rs1799722 and rs7675300	0.034	0.965	1.034 (0.232–4.605)

B; regression coefficient, OR; odds ratio, CI; confidence interval.

## Discussion

The current study provides valuable insights into the genetic determinants influencing ACEI-induced cough within a multi-ethnic population. We validated the suggested variants and examined their effect on patients complaining of this side effect. We further evaluated the effect of one common variant in the *ACE* gene on ACE plasma levels.

ACEIs are commonly prescribed for treating hypertension and other cardiovascular diseases, but a persistent dry cough is a clinically important side effect affecting up to 35% of patients. This side effect is dose-independent, unpredictable, and represents a unique reaction in patients who are predisposed, and it can occur at any time from 1 week up to a year after starting the medication. Although the cough is typically mild to moderate in severity, there are cases where it becomes severe enough to require discontinuation of the treatment and switching to alternatives, such as angiotensin receptor blockers (ARBs) (Borghi et al., 2023; Vegter and De Jong-van den Berg, 2010; Zamora and Parodi, 2011).

Diagnosing ACEI-induced cough is difficult due to the absence of physical biomarkers, and its exact mechanism remains unclear. However, it is thought that BK and substance P play vital roles. ACE normally metabolizes BK, and ACEIs lead to BK accumulation in the respiratory tract, which binds to BDKRB2 receptors, causing histamine release, resulting in bronchospasm and cough. The reason why only some ACEIs users experience cough remains to be determined. However, research suggests that genetic predispositions, including *ACE* I/D, *BDKRB2*, and *KCNIP4* variants, may contribute. This suggests that multiple mechanisms may be involved rather than a single factor (Ancion et al., 2019; Mosley et al., 2016; Yılmaz, 2019; Zamora and Parodi, 2011). The relationship between these gene variants and susceptibility to ACEI-induced cough has been studied in limited studies.

A meta-analysis of the *ACE* rs1799752 I/D variant found that individuals with the I/I genotype had a significant association with ACE inhibitor-induced cough, particularly in East Asian populations (Nishio et al., 2011). In contrast, other studies reported no link between rs1799752 and ACEI-induced cough in African and Caucasian populations (Moholisa et al., 2013; Rohman et al., 2018). Additionally, regarding the *BDKRB2* rs1799722 (C>T) SNP, Mukae and coworkers investigated the relationship between the T/T genotype and ACEI-induced cough, particularly in Japanese females (Mukae et al., 2000). Their later study further supported a strong association between the T/T genotype and the T allele with ACEI-induced cough, suggesting a genetic predisposition to this side effect in the Japanese population (Mukae et al., 2002). However, there was no association between rs1799722 (C>T) and ACEI-induced cough in Korean and South African populations (Moholisa et al., 2013; Woo et al., 2009). Furthermore, a Genome-Wide Association Study (GWAS) identified a connection between the rs7675300 (C>A), rs1495509 (T>C), rs7661530 (T>C), and rs16870989 (T>A) SNPs on the *KCNIP4* gene and ACEI-induced cough (Mosley et al., 2016). In contrast, a separate GWAS on a Swedish population found no association between these SNPs and ACEI-induced cough (Hallberg et al., 2017).

Considering the contradictory findings across various studies regarding the association between these variants and ACEI-induced cough, and recognizing the lack of research on this relationship in Middle Eastern countries and populations, we aimed to investigate the relationship between these variants and ACEI-induced cough within the UAE population. We also investigated the difference in ACE plasma levels between the two study groups and the effect of *ACE* rs1799752 I/D genotypes on ACE plasma levels in this population. Notably, this study is the first to include all these variants in a single analysis. In the present study, we identified that four variants, rs1799722 (C>T) on *BDKRB2*, rs7675300 (C>A), rs1495509 (T>C), and rs16870989 (T>A) on *KCNIP4,* showed no statistically significant association with ACEI-induced cough. However, noteworthy exceptions emerged: the I/D genotype of rs1799752 (I/D) on *ACE* was significantly associated with an increased risk of ACEI-induced cough following gender adjustment in the over-dominant model. Furthermore, we observed a significant association between the T/T genotype of rs7661530 (T>C) in *KCNIP4* and ACEI-induced cough in both codominant and recessive models, both before and after adjusting for gender.

Moreover, the current study’s findings reveal a significant difference in ACE plasma levels between the cough and non-cough groups, with the non-cough group having elevated ACE levels. This result suggests a potential association between reduced ACE plasma levels and the occurrence of cough (p < 0.01). However, a unique pattern emerged when we examined ACE plasma levels in relation to the *ACE* rs1799752 (I/D) genotype (D/D, I/D, I/I) and cough status. In particular, within the I/D genotype, the non-cough group exhibited significantly higher ACE levels than the cough group (p < 0.01). In contrast, no significant differences were observed between the cough and non-cough groups for the D/D and I/I genotypes. This suggests that the I/D genotype may be a significant factor in explaining the variations in ACE plasma levels associated with cough status (cough and non-cough). This noteworthy discovery in the I/D group may explain why this genotype was the only one previously linked to cough in our study, possibly suggesting a molecular relationship between the incidence of cough in this genotype and ACE plasma levels within the study population.

Furthermore, in our stratified analysis by ethnicity, we observed significant differences in the distribution of genotypes among ethnic groups, particularly for the *ACE* rs1799752 (I/D) and *KCNIP4* variants (rs7675300 (C>A), rs1495509 (T>C), rs16870989 (T>A)). The Arab population showed a consistently higher frequency of the D/D genotype in *ACE* rs1799752 and the C/C and T/T genotypes in *KCNIP4* rs7675300 and rs1495509 variants respectively, whereas the East Asian and Indian groups displayed higher frequencies of alternative genotypes. These differences highlight the genetic diversity across ethnicities and may partially explain variations in ACEI-induced cough among populations. The findings support the importance of considering population background in pharmacogenomic research and future clinical implementation. Further large-scale, prospective studies with balanced representation of ethnic groups are needed to validate these associations and explore their clinical significance.

Additionally, results showed that variants of the *KCNIP4*; rs7675300 (C>A), rs1495509 (T>C), rs7661530 (T>C), and rs1687089 (T>A) are in high LD with each other, indicating they are often inherited together within the population studied. This strong LD suggests the presence of haplotype blocks, and based on this association, we have constructed haplotypes further to investigate the potential implications for ACEI-induced cough. However, the global association p = 0.38 indicates that the haplotypes, when considered collectively, did not exhibit a statistically significant correlation with ACEI-induced cough. Furthermore, we assessed the combined effect of *ACE*; rs1799752 (I/D), *BDKRB2*; rs1799722 (C>T), and *KCNIP4*; rs7675300 (C>A) on ACEI-induced cough. The variants interaction analysis did not reveal any statistically significant associations indicating a lack of strong evidence for any meaningful effect of these variant’s interactions on ACEI-induced cough.

However, adjustments for additional covariates such as age, smoking status, comorbidities, and ACEIs type using multivariable models were not feasible due to the small sample size. Additionally, no multiple testing correction was applied, therefore, these results should be interpreted with appropriate caution. These limitations are acknowledged to provide a balanced context for our findings.

Finally, this study represents the first investigation of the association between these six genetic variants and ACEI-induced cough in an Arab population and in the Middle East. However, the findings require replication in independent cohorts to confirm their reproducibility and generalizability. Future studies involving larger and ethnically diverse Middle Eastern populations are essential to validate these pharmacogenomic associations. Such replication will help to establish robust genetic markers for predicting ACEI-induced cough and guide personalized treatment strategies in different populations. In addition, further functional assay studies are needed to explore the potential impact of the *ACE* rs1799752 (I/D) variant on ACE expression and activity to clarify the mechanistic role of this variant and enhance the clinical interpretation of our findings.

## Conclusion

This study provides valuable insights into the genetic susceptibility to ACEI-induced cough by examining variants in *ACE*, *BDKRB2*, and *KCNIP4*. The significant associations identified between the I/D genotype of *ACE*: rs1799752 I/D, and the T/T genotype of *KCNIP4*, rs7661530 (T>C) with ACEI-induced cough emphasize the role of genetic factors in this side effect. Furthermore, the observation that total ACE plasma levels were significantly higher in the non-cough group, particularly among individuals with the I/D genotype, underscores the potential effect of ACE levels on cough development. These findings underscore the significance of genetic factors and ACE plasma levels in determining the risk of ACEI-induced cough, which may inform personalized treatment strategies for patients at higher risk.

### Limitations

The current study has limitations that should be acknowledged. First, the relatively small sample size may limit the generalizability of the findings, as a larger cohort would likely yield more robust and statistically reliable results. Second, the study focused primarily on perindopril and lisinopril, as these were the most commonly prescribed ACE inhibitors at the recruitment sites. This focus may restrict the applicability of the findings to other ACEIs, such as enalapril or ramipril, which were underrepresented in the cohort.

## Data Availability

The data presented in this study are not publicly available due to participant privacy concerns and data sharing restrictions set by the institutional ethics board. De-identified data may be made available upon reasonable request to the corresponding author, subject to ethical approval.
