# Supplementary material for: Pharmacogenomic biomarkers of ACE inhibitor–induced cough in a multi-ethnic UAE cohort
Source: Front Pharmacol. 2025 Sep 25;16:1655617. doi: 10.3389/fphar.2025.1655617 (PMC12508485; doi:10.3389/fphar.2025.1655617)
Supplement: Supplementary file 1 [file Table1.docx]

**Supplementary Material**

**Pharmacogenomic Biomarkers of ACE Inhibitor–Induced Cough in a Multi-Ethnic UAE Cohort**

Sahar M. Altoum^1^, Zeina N. Al-Mahayri^2^, Lubna Q. Khasawneh^1^, Mais N. Alqasrawi^1^, Lilas Dabaghie^1^, Dana Hamza^1^, Bassam R. Ali^1*^

**Table S1:** Hardy-Weinberg Equilibrium (HWE) analysis for the studied variants.

| **Gene** | **Variant** | **Chi^2^ in (Cough)** | **P value (Cough)** | **Chi^2^ in**  **(Non-cough)** | **P value**  **(Non-cough)** | **Chi^2^ in both** | **P value in both** |
| --- | --- | --- | --- | --- | --- | --- | --- |
| *ACE* | rs1799752 | 0.087 | 1 | 7.529 | 0.148 | 4.272 | 0.29 |
| *BDKRB2* | rs1799722 | 1.631 | 0.797 | 0.303 | 0.923 | 0.075 | 1 |
| *KCNIP4* | rs7675300 | 0.478 | 1 | 0.249 | 1 | 0.938 | 0.73 |
|  | rs1495509 | 0.719 | 0.846 | 0.249 | 1 | 0.959 | 0.723 |
|  | rs7661530 | 5.734 | 0.241 | 1.052 | 0.766 | 6.138 | 0.197 |
|  | rs16870989 | 0.079 | 1 | 0.249 | 1 | 0.464 | 0.853 |

p<0.05 was considered significant.

| **rs ID (gene)** | **Genotype** |  | **Ethnicity** | | | | | **Total** | **P value** |
| --- | --- | --- | --- | --- | --- | --- | --- | --- | --- |
|  |  |  | African | Arab | East Asian | Indian | Others |  |  |
| rs1799752  (*ACE*) | D/D | N | 1 | 33 | 4 | 5 | 1 | 44 | **<0.01** |
|  |  | Adjusted Residual | 0.3 | 4.4 | -0.8 | -4.4 | 1.2 | xx |  |
|  | I/D | N | 0 | 16 | 4 | 21 | 0 | 41 |  |
|  |  | Adjusted Residual | -1.1 | -1.7 | -0.6 | 2.7 | -0.8 | xx |  |
|  | I/I | N | 1 | 4 | 5 | 12 | 0 | 22 |  |
|  |  | Adjusted Residual | 1.0 | -3.3 | 1.7 | 2.1 | -0.5 | xx |  |
| Total | | N | 2 | 53 | 13 | 38 | 1 | 107 |  |

**Table S2:** The distribution of *ACE* rs1799752 (I/D) variant within ethnicities.

N: Number of patients.

p<0.05 was considered significant.

| **rs ID (gene)** | **Genotype** |  | **Ethnicity** | | | | | **Total** | **P value** |
| --- | --- | --- | --- | --- | --- | --- | --- | --- | --- |
|  |  |  | African | Arab | East Asian | Indian | Others |  |  |
| rs1799722  *(BDKRBB2)* | C/C | N | 1 | 17 | 2 | 17 | 0 | 37 | 0.15 |
|  | C/T | N | 0 | 29 | 9 | 15 | 0 | 53 |  |
|  | T/T | N | 1 | 7 | 2 | 6 | 1 | 17 |  |
| Total | | N | 2 | 53 | 13 | 38 | 1 | 107 |  |

**Table S3:** The distribution of *BDKRB2* rs1799722 (C>T) variant within ethnicities

N: Number of patients.

p<0.05 was considered significant.

**Table S4:** The distribution of *KCNIP4* rs7675300 (C>A) variant within ethnicities

| **rs ID (gene)** | **Genotype** |  | **Ethnicity** | | | | | **Total** | **P value** |
| --- | --- | --- | --- | --- | --- | --- | --- | --- | --- |
|  |  |  | African | Arab | East Asian | Indian | Others |  |  |
| rs7675300  (*KCNIP4*) | A/A | N | 0 | 5 | 2 | 7 | 0 | 14 | **0.04** |
|  |  | Adjusted Residual | -0.6 | -1.1 | 3 | 1.2 | -0.4 | xx |  |
|  | C/A | N | 2 | 15 | 6 | 20 | 0 | 43 |  |
|  |  | Adjusted Residual | 1.7 | -2.5 | 0.5 | 1.9 | -0.8 | xx |  |
|  | C/C | N | 0 | 33 | 5 | 11 | 1 | 50 |  |
|  |  | Adjusted Residual | -1.3 | 3.2 | -0.6 | -2.7 | 1.1 | xx |  |
| Total | | N | 2 | 53 | 13 | 38 | 1 | 107 |  |

N: Number of patients.

p<0.05 was considered significant.

**Table S5:** The distribution of *KCNIP4* rs1495509 (T>C) variant within ethnicities

| **rs ID (gene)** | **Genotype** |  | **Ethnicity** | | | | | **Total** | **P value** |
| --- | --- | --- | --- | --- | --- | --- | --- | --- | --- |
|  |  |  | African | Arab | East Asian | Indian | Others |  |  |
| rs1495509 (*KCNIP4*) | C/C | N | 0 | 5 | 2 | 6 | 0 | 13 | **0.04** |
|  |  | Adjusted Residual | -0.5 | -0.9 | 0.4 | 0.9 | -0.4 | xx |  |
|  | T/C | N | 2 | 15 | 4 | 21 | 0 | 42 |  |
|  |  | Adjusted Residual | 1.8 | -2.3 | -0.7 | 2.5 | -0.8 | xx |  |
|  | T/T | N | 0 | 33 | 7 | 11 | 1 | 52 |  |
|  |  | Adjusted Residual | -1.4 | 2.8 | 0.4 | -3.0 | 1.0 | xx |  |
| Total | | N | 2 | 53 | 13 | 38 | 1 | 107 |  |

N: Number of patients.

p<0.05 was considered significant.

**Table S6:** The distribution of *KCNIP4* rs7661530 (T>C) variant within ethnicities.

N: Number of patients.

p<0.05 was considered significant.

| **rs ID (gene)** | **Genotype** |  | **Ethnicity** | | | | | **Total** | **P value** |
| --- | --- | --- | --- | --- | --- | --- | --- | --- | --- |
|  |  |  | African | Arab | East Asian | Indian | Others |  | 0.23 |
| rs7661530  (*KCNIP4*) | C/C | N | 1 | 32 | 9 | 13 | 1 | 56 |  |
|  | T/C | N | 1 | 15 | 3 | 16 | 0 | 35 |  |
|  | T/T | N | 0 | 6 | 1 | 9 | 0 | 16 |  |
| Total | | N | 2 | 53 | 13 | 38 | 1 | 107 |  |

**Table S7:** The distribution of *KCNIP4* rs16870989 (T>A) variant within ethnicities.

| **rs ID (gene)** | **Genotype** |  | **Ethnicity** | | | | | **Total** | **P value** |
| --- | --- | --- | --- | --- | --- | --- | --- | --- | --- |
|  |  |  | African | Arab | East Asian | Indian | Others |  |  |
| rs16870989  (*KCNIP4*) | A/A | N | 0 | 5 | 2 | 6 | 0 | 13 | **0.04** |
|  |  | Adjusted Residual | -0.5 | -0.9 | 0.4 | 0.9 | -0.4 | xx |  |
|  | T/A | N | 2 | 15 | 6 | 21 | 0 | 44 |  |
|  |  | Adjusted Residual | 1.7 | -2.7 | 0.4 | 2.2 | -0.8 | xx |  |
|  | T/T | N | 0 | 33 | 5 | 11 | 1 | 50 |  |
|  |  | Adjusted Residual | -1.3 | 3.2 | -0.6 | -2.7 | 1.1 | xx |  |
| Total | | N | 2 | 53 | 13 | 38 | 1 | 107 |  |

N: Number of patients.

p<0.05 was considered significant.
